# Isothermal cross-priming amplification for field-deployable norovirus surveillance: a cost-effective and rapid solution

**DOI:** 10.1128/spectrum.01684-26

**Published:** 2026-09-04

**Authors:** Ran Jia, Lijuan Lu, Xunhua Zhu, Lingfeng Cao, Liyun Su, Huaqing Zhong, Menghua Xu, Pengcheng Liu, Yanqiong Zhou, Jin Xu

**Affiliations:** 1 Department of Clinical Laboratory, Children's Hospital of Fudan University, National Children's Medical Center 145601 , Shanghai, China; 2 Ustar Biotechnologies (Hangzhou) Ltd, Hangzhou, Zhejiang, China; US Food and Drug Administration, Silver Spring, Maryland, USA

**Keywords:** norovirus, isothermal amplification, cross-priming amplification

## Abstract

Norovirus remains a leading cause of foodborne gastroenteritis globally. While isothermal amplification technologies offer advantages over traditional reverse transcription quantitative PCR (RT-qPCR) in terms of speed and portability, there is a lack of domestically developed isothermal amplification systems for norovirus in China. Using a domestic patented cross-priming amplification (CPA) technology, we established CPA systems targeting the predominant norovirus genotypes GII.3, GII.4, and GII.17. The systems were evaluated using 224 fecal samples with confirmed genotypes via sequencing. Our lab-developed CPA (Lab-CPA) systems demonstrated high sensitivity, detecting 98.0% (49/50) of GII.3, 100% (80/80) of GII.4, and 96.9% (31/32) of GII.17 samples, with 100% specificity across all genotypes. Compared to certified clinical RT-qPCR kits, the total percent agreement was 98.7%, 99.3%, and 96.9% for GII.3, GII.4, and GII.17, respectively, accompanied by a significant linear correlation in threshold times. Crucially, the Lab-CPA systems reduced processing time by 44–60 min compared to RT-qPCR. Successful integration into the commercial EasyNAT platform using all-in-one cartridges further streamlined the workflow while maintaining accuracy. Our CPA systems offer a rapid, cost-effective, and streamlined diagnostic solution, ideally suited for point-of-care testing in resource-limited primary healthcare environments.

**IMPORTANCE:**

Norovirus remains a critical public health threat, driving demand for rapid, deployable diagnostics. While isothermal amplification offers theoretical advantages over reverse transcription quantitative PCR, the absence of domestically developed systems in China has created a reliance on imported assays, limiting point-of-care testing capabilities. This study addresses that gap by establishing a novel cross-priming amplification system tailored to China’s predominant norovirus genotypes. Achieving high sensitivity/specificity and significantly reducing processing time, this work validates a robust, field-adaptable alternative to conventional methods. Furthermore, successful integration with the commercial EasyNAT platform demonstrates immediate translational potential. By providing a cost-effective, high-performance domestic solution, these findings significantly enhance decentralized surveillance and emergency response capacity for norovirus infections in resource-limited settings.

## INTRODUCTION

Norovirus, a highly infectious non-enveloped RNA virus belonging to the *Caliciviridae* family, is a leading cause of foodborne gastroenteritis worldwide (1). Requiring only a small amount of particles (10–100 particles) to establish infection, norovirus results in approximately 700 million cases and 220,000 deaths annually, mainly affecting children under 5 (2, 3). While most norovirus infections typically manifest as self-limiting vomiting and diarrhea resolving within 72 hours (2), vulnerable populations (elderly, immunocompromised individuals, and newborns) face elevated risks of severe outcomes (2), necessitating prompt diagnosis and surveillance.

Norovirus is classified into 10 genogroups (GI–GX) based on variations in the VP1 capsid protein. In China, GII.4 and GII.3 have historically dominated, accounting for over 90% of outbreaks and sporadic cases in China in the past decade (4, 5). However, recent surveillance, including our own epidemiological investigation (6), indicates a significant shift, with emergent GII.17 strains superseding GII.4 as the predominant genotype across Europe (7–9), the United States (10, 11), and China (12). Therefore, to ensure broad coverage and clinical utility, this study specifically targeted the three most prevalent genotypes: GII.3, GII.4, and GII.17. Crucially, these genotypes exhibit distinct clinical profiles. While GII.4 circulates broadly across all age groups, causing typical gastroenteritis (13), GII.17 preferentially targets older children and is associated with severe, projectile vomiting and heightened dehydration risk (9, 11, 14). In contrast, GII.3 is predominantly detected in children <24 months (15), often linked to diarrhea with less frequent vomiting (16). This divergence underscores the necessity of precise genotyping for targeted clinical intervention.

Current diagnostic procedures for norovirus infection rely heavily on reverse transcription quantitative PCR (RT-qPCR). However, the typical workflow of this technique requires multiple steps, including nucleic acid extraction, reverse transcription, and real-time PCR cycling, which is time-consuming (~2 to 3 h) and lacks portability (17). To address these limitations, several isothermal amplification technologies have emerged as promising alternatives, including loop-mediated isothermal amplification (LAMP) and recombinase polymerase amplification (RPA) (18–20). As reported, LAMP is capable of achieving 10⁹- to 10¹⁰-fold amplification within 15–60 min under constant temperature (~65°C) (18, 19) and has been shown to yield detection rates that are comparable to RT-qPCR for specific pathogens (18). RPA is also a highly specific and sensitive isothermal amplification technique (~40°C), and it has been successfully adopted for the detection of various target pathogens (20). However, these technologies are protected by foreign patents, resulting in higher costs and deployment constraints in our country.

Cross-priming amplification (CPA) is a domestically patented isothermal technology that has the potential to substitute LAMP and RPA in our country. This technology utilizes Bst DNA polymerase with strand displacement specificity and betaine to efficiently amplify the target sequence at a constant temperature (~60°C) within 40–60 min (21). This technology has been fully integrated into the commercially available EasyNAT molecular diagnostic platform (21–23), which is designed for point-of-care testing (POCT). Characterized by rapid on-site detection of patient samples, POCT requires portable analysis and immediate result readout (22). To meet these demands, the EasyNAT platform seamlessly combines magnetic bead-based nucleic acid extraction and CPA amplification within a single reaction chamber, thereby minimizing hands-on time and reducing contamination risk (24). So far, it has demonstrated strong clinical performance in comparison to RT-qPCR across multiple targets, including *Mycobacterium tuberculosis*, SARS-CoV-2, *Chlamydia trachomatis, Neisseria gonorrhoeae*, and *Mycoplasma pneumoniae* (21, 24, 25). Hence, the combination of operational simplicity, minimal infrastructure requirements, adequate accuracy, and rapid processing time positions EasyNAT as a practical alternative to lab-bound RT-qPCR assays for frontline screening and near-patient diagnosis.

Building on the untapped potential of CPA technology for norovirus testing, this study aimed to develop and validate a novel CPA assay enabling both rapid detection and concurrent genotyping. We demonstrated that our CPA systems greatly simplify experimental workflows while yielding results highly concordant with reference RT-qPCR, rendering it a reliable diagnostic tool for norovirus identification in a broad spectrum of clinical scenarios.

## MATERIALS AND METHODS

### Study design

From January 2019 to January 2024, fecal specimens were prospectively collected from patients presenting with suspected norovirus infection. Eligible participants met the case definition for acute gastroenteritis, with clinical manifestations comprising vomiting, diarrhea, abdominal cramping, and/or low-grade fever. All samples were surplus de-identified specimens remaining after routine diagnostic workflows.

All of the clinical specimen processing and detection was performed within the accredited biosafety level 2 laboratory at the Clinical Laboratory Center, Children’s Hospital of Fudan University. All operations were conducted in class II biosafety cabinets with standardized personal protective equipment. All biohazardous waste was autoclaved (121°C, 30 min) before disposal, and surfaces were routinely decontaminated following institutional biosafety protocols.

### RT-qPCR for norovirus detection

The samples were analyzed for norovirus using a clinically validated RT-qPCR system (Land Medical, China) implemented on Applied Biosystems 7500 Real-Time PCR platform (ABI 7500, USA). This two-step integrated protocol combines reverse transcriptase-mediated cDNA synthesis (42°C/30 min) with the subsequent qPCR process (95°C/10 s, 60°C/1 min, 40 cycles). Quantitative interpretation was performed by exporting threshold cycle (Ct) values through the manufacturer’s proprietary analysis software. The actual time (Tq) required to reach the Ct in RT-qPCR is calculated based on the thermal cycling parameters of the instrument.

### Norovirus genotyping

The fecal samples underwent nucleic acid extraction via a magnetic bead-based extraction method (Daan Gene, China), followed by cDNA synthesis employing the PrimeScript reverse transcription system (Takara, Japan). Amplification was conducted with VP1-specific primers (G2SKF/G2SKR) using Premix Taq polymerase (Takara) as previously described (26). Amplicons were sequenced through Sanger sequencing (Sangon Biotech, China), with subsequent genotyping achieved via alignment against norovirus reference strains in GenBank.

### Phylogenetic analysis

Our CPA detection systems focused specifically on the GII.3, GII.4, and GII.17 genotypes due to their epidemiological predominance (6). Representative strains of the three genotypes were chosen for phylogenetic reconstruction. Using the MUSCLE algorithm, the nucleotide sequences of the VP1 region derived from this study were aligned with reference sequences obtained from GenBank. Phylogenetic trees were constructed using the neighbor-joining method, and branch support was assessed using 1,000 bootstrap iterations. All phylogenetic analyses were performed using MEGA software (version 11).

### Lab-developed CPA assay for norovirus

Primers were designed based on the specific principles of CPA, which requires a set of six primers to initiate strand displacement and exponential amplification (27). The VP1 gene was specifically targeted, as it contains conserved regions suitable for genotyping. To further identify the conserved motifs, we aligned multiple reference sequences of the GII.3, GII.4, and GII.17 genotypes obtained from GenBank. Based on these alignments, genotype-specific primer sets were designed using SnapGene software (version 2.3.2) (Table 1; Table S1). According to the principle of CPA, target melting temperature (Tm) values were set according to primer function: cross primers (CPR and CPF) and the probe-bearing primer (AMP2) were designed with Tm typically around 65°C to ensure stable target hybridization at the 58°C reaction temperature, while displacement primers (FB and RB) and the internal primer (IP1) were set to Tm values around 55°C, slightly below the reaction temperature, to facilitate strand displacement. To minimize secondary structure-mediated inhibition, primers were screened for hairpin formation and primer-dimer potential using the IDT OligoAnalyzer tool (https://sg.idtdna.com/pages/tools/oligoanalyzer). Notably, the universal stem-loop adapter of the AMP2 probe, which forms an intentional secondary structure, was assessed as part of the intact probe construct rather than as an isolated oligonucleotide. The primers were synthesized by Sangon Biotech.

**TABLE 1:** Primers and probes of the CPA system targeting GII.3, GII.4, and GII.17 subtypes of norovirus^*a*^

Targets	Primer name	Sequence (5′–3′)
GII.3	IP1	CCGGCGGCACCATCATTAG
	AMP2	5′-FAM-CCGAGCGTTACGCTCGG/iBHQ1dT/GGCCAGCAAAATATAATTGATCCCTGGA
	CPR	GATAGCAGCACCCCTCACCGGTGCTTGCACAAAATTATTCAT
	CPF	GCCTCATTGTTGATCTCTGGGACCGAATGACGCCACCCCAT
	RB	GGACACTGTGAACTCACCACCA
	FB	CGTTGTGATGAGTGGCGT
GII.4	IP1	GCCATCAGTTGTGCATCTGCCGTT
	AMP2	5′-FAM-CCGAGCGTTACGCTCGG/iBHQ1dT/AACTACACTATGAATTTGGCTTCTCAA
	CPR	GTCACTCACATCACAGGTAGTCACTCTGTTGGATCATAGTTGTTCCAATT
	CPF	GTTGGGTGGTGCCTAGAAGCAAGTGCATTTGTCGTTCAACCACA
	RB	AGTTCCTAGAGGGGCTGGGATTTCT
	FB	AAGTTGTTTACAGGTCCCAGC
GII.17	IP1	TCAATTATATTATTTTGGCCAG
	AMP2	5′-FAM-CCGAGCGTTACGCTCGG/iBHQ1dT/AATGGAGAGTTCACAGTGTCACCC
	CPR	AACAAATTTTGTRCAAGCTTTARTAAAATTTCTCCAGGAGAG
	CPF	ATCCAGGGGTCAATTAACCAGTTGCGGGCGCAGC
	RB	ATCAGGGCCCAACTCTA
	FB	GAGACCCTTCCCCTAG

^*a*^ AMP2, AMP molecular beacon; CPF, crossing primer forward; CPR, crossing primer reverse; FB, forward bump; IP1, internal primer; RB, reverse bump.

For the lab-developed CPA (Lab-CPA) workflow, nucleic acids were extracted from fecal samples using a magnetic bead-based extraction method (Daan Gene) and then subjected to CPA amplification on the ABI 7500 System under optimized conditions: 58°C for 1 h with continuous fluorescence monitoring (Fig. 1A). Positive reactions generated a time-threshold value (Tt), defined as the time required for the fluorescence signal to surpass a precalibrated threshold.

**Fig 1:**
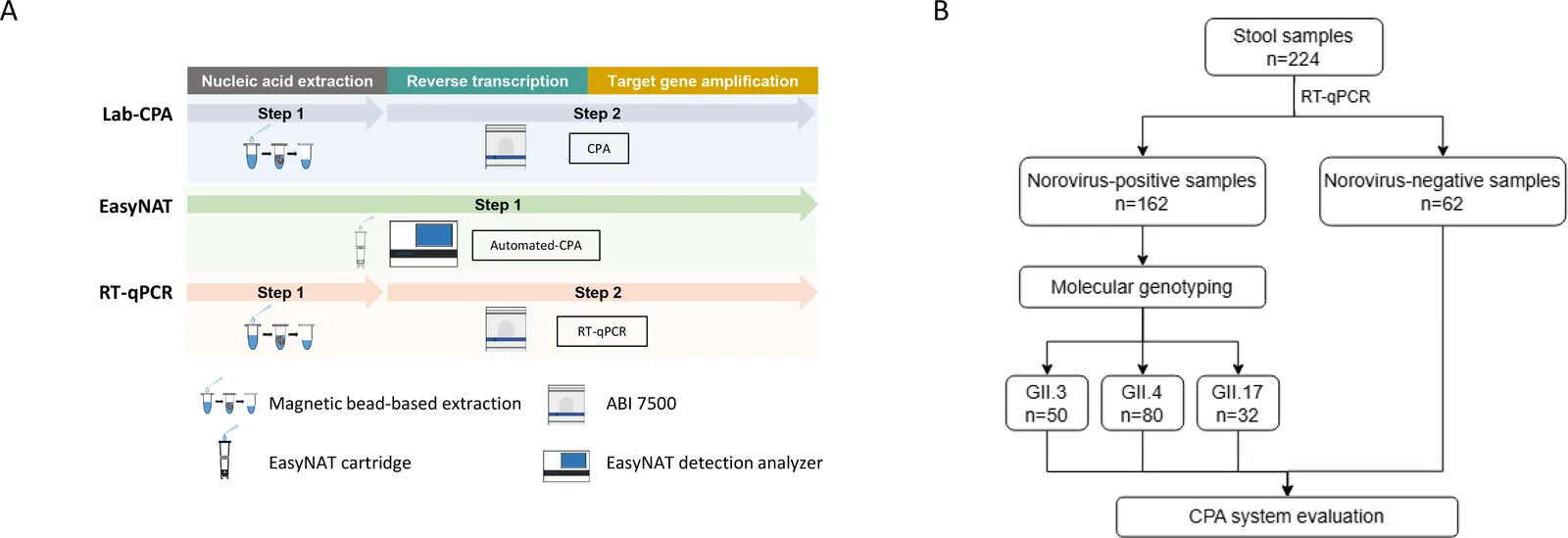
Experimental workflow. (**A**) Schematic comparison of the operational workflows for Lab-CPA, EasyNAT, and conventional RT-qPCR. (**B**) Flowchart illustrating the sample recruitment and screening process for the validation of the CPA systems. Lab-CPA, lab-developed CPA.

### CPA assay based on the EasyNAT detection system

The Lab-CPA systems were integrated into the EasyNAT platform, utilizing a fully automated cartridge that consolidates magnetic bead-based nucleic acid extraction and CPA amplification. The cartridge architecture features a main chamber and two dependent side chambers preloaded with genotype-specific CPA primers/probes, Bst DNA polymerase, and reaction buffer (see Fig. 3A). Upon loading the fecal sample, the EasyNAT analyzer executes a predefined thermal-kinetic program to drive the integrated workflow. Amplification kinetics are monitored in real time, yielding a time-to-threshold value (*T*_E_) for quantitative analysis.

### Statistical method

Continuous data were presented as medians with interquartile ranges (IQRs); categorical data were expressed as medians with percentages. The chi-square test or Fisher’s exact test was used to compare the proportions of categorical variables. In the analysis of continuous data for two-group comparisons, normally distributed variables with equal variances were analyzed using the unpaired Student’s *t*-test, while the Welch-adjusted *t*-test was applied to accommodate unequal variances. Non-normally distributed variables were assessed via the Mann-Whitney *U* test. Statistical analyses were performed using GraphPad Prism software (version 10) and IBM SPSS Statistics software (version 23). Two-sided *P* values of <0.05 were considered statistically significant.

To evaluate the agreement between two methods, we used Cohen’s kappa coefficient (*κ*). Cohen’s kappa measures the degree of agreement between two raters classifying items into mutually exclusive categories while correcting for agreement that would be expected by chance alone (28, 29). The kappa coefficient was interpreted according to the criteria proposed by Landis and Koch (30): ≤0.20 indicates slight agreement; 0.21–0.40, fair; 0.41–0.60, moderate; 0.61–0.80, substantial; and 0.81–1.00, almost perfect agreement. All kappa analyses were performed using IBM SPSS Statistics software (version 23).

## RESULTS

### The Lab-CPA systems perform adequately in detecting GII.3, GII.4, and GII.17

First, we established three independent CPA systems that target the prevailing GII.3, GII.4, and GII.17 genotypes by leveraging the existing laboratory infrastructure (Lab-CPA systems) (Fig. 1A). Primer sequences are detailed in Table 1. For validation, a prospective cohort of 224 fecal specimens was collected from patients, comprising 162 norovirus-positive samples (50 GII.3-positive samples, 80 GII.4-positive samples, and 32 GII.17-positive samples) and 62 norovirus-negative samples (Fig. 1B). A phylogenetic tree was constructed based on sequence alignments of the target strains and reference sequences retrieved from GenBank (Fig. S1). The resulting topology revealed that the viral strains selected for validation are phylogenetically distinct and segregate into the three targeted genotypes—GII.3, GII.4, and GII.17—thereby confirming the accuracy of our genotyping result.

For the validation of the GII.3 Lab-CPA system, a total of 102 samples were tested. It correctly identified 49 of 50 GII.3-positive samples and all 52 GII.3-negative samples, yielding a positive predictive value (PPV) of 100.0% (49/49) and a negative predictive value (NPV) of 98.1% (52/53), a sensitivity of 98.0% (49/50), and a specificity of 100.0% (52/52) (Table 2). The GII.4 Lab-CPA system demonstrated excellent performance across all 164 samples (80 GII.4 positive, 84 GII.4 negative), achieving 100% for all diagnostic metrics (Table 2). The GII.17 Lab-CPA system was evaluated using 80 samples, identifying 31 of 32 positive cases and all 48 negative controls. This resulted in a PPV of 100.0% (31/31), an NPV of 98.0% (48/49), a sensitivity of 96.9% (31/32), and a specificity of 100.0% (48/48) (Table 2). Collectively, these data confirm that the three Lab-CPA systems exhibit robust and reliable performance for detecting GII.3, GII.4, and GII.17.

**TABLE 2:** Detection results of Lab-CPA systems^*a*^

Sample types	Sample number	Lab-CPA results		PPV (%)	NPV (%)	Sensitivity (%)	Specificity (%)
		Positive	Negative				
GII.3 Lab-CPA system							
GII.3 positive (*n* = 50)							
Norovirus positive (GII.3)	50	49	1				
GII.3 negative (*n* = 52)							
Norovirus positive (non-GII.3)	12	0	12				
Norovirus negative	28	0	28				
NTC	12	0	12				
Overall	102	49	53	100.0	98.1	98.0	100.0
GII.4 Lab-CPA system							
GII.4 positive (*n* = 80)							
Norovirus positive (GII.4)	80	80	0				
GII.4 negative (*n* = 84)							
Norovirus positive (non-GII.4)	14	0	14				
Norovirus negative	62	0	62				
NTC	8	0	8				
Overall	164	80	84	100.0	100.0	100.0	100.0
GII.17 Lab-CPA system							
GII.17 positive (*n* = 32)							
Norovirus positive (GII.17)	32	31	1				
GII.17 negative (*n* = 48)							
Norovirus positive (non-GII.17)	8	0	8				
Norovirus negative	32	0	32				
NTC	8	0	8				
Overall	80	31	49	100.0	98.0	96.9	100.0

^*a*^ NPV, negative predictive value; NTC, no template control; PPV, positive predictive value.

### **‌**The Lab-CPA systems yield accuracy comparable to RT-qPCR but are more time efficient

‌We then compared the performance of the Lab-CPA systems with the RT-qPCR assay (Table 3). For the GII.3 Lab-CPA system, 78 samples were tested, with the positive percent agreement (PPA), negative percent agreement (NPA), and total percent agreement (TPA) being 98% (49/50), 100% (28/28), and 98.7% (77/78), respectively (Table 3). A total of 142 samples were tested for the GII.4 Lab-CPA system validation, presenting a PPA of 100% (79/79), an NPA of 98.4% (62/63), and a TPA of 99.3% (141/142) (Table 3). The GII.17 Lab-CPA system was evaluated using 64 samples, yielding a PPA of 96.8% (30/31), an NPA of 97.0% (32/33), and a TPA of 96.9% (62/64) (Table 3). Kappa analysis confirmed substantial agreement of Lab-CPA systems with the RT-qPCR reference (*κ* = 0.972, 0.986, and 0.937 for GII.3, GII.4, and GII.17, respectively).

**TABLE 3:** Performance of the Lab-CPA systems compared with RT-qPCR^*a*^

		RT-qPCR		PPA (%)	NPA (%)	TPA (%)
		Positive	Negative			
GII.3 Lab-CPA	Positive	49	0	98.0	100.0	98.7
	Negative	1	28			
GII.4 Lab-CPA	Positive	79	1	100.0	98.4	99.3
	Negative	0	62			
GII.17 Lab-CPA	Positive	30	1	96.8	97.0	96.9
	Negative	1	32			

^*a*^ NPA, negative percent agreement; PPA, positive percent agreement; TPA, total percent agreement.

To further investigate the consistency between the Lab-CPA systems and the RT-qPCR assay, we analyzed the correlation of their threshold times. Given that the Lab-CPA systems could output a threshold time (Tt) directly, the RT-qPCR cycle threshold (Ct) values were converted into equivalent reaction times (Tq) on the basis of the thermal cycling parameters. As a result, a significant positive linear correlation was observed between Tt and Tq for all three genotypes (GII.3: *r* = 0.584, *P* = 0.0004; GII.4: *r* = 0.487, *P* = 0.0026; GII.17: *r* = 0.586, *P* = 0.0016; Fig. 2). This correlation suggests that the amplification kinetics of the Lab-CPA systems closely parallel those of the reference RT-qPCR method. Crucially, the Lab-CPA systems shortened the process by medians of 53 min (IQR 47–58), 60 min (IQR 54–66), and 44 min (IQR 41–51) for GII.3, GII.4, and GII.17, respectively. These findings underscore that our Lab-CPA systems combine diagnostic accuracy comparable to gold-standard RT-qPCR with markedly improved operational efficiency.

**Fig 2:**
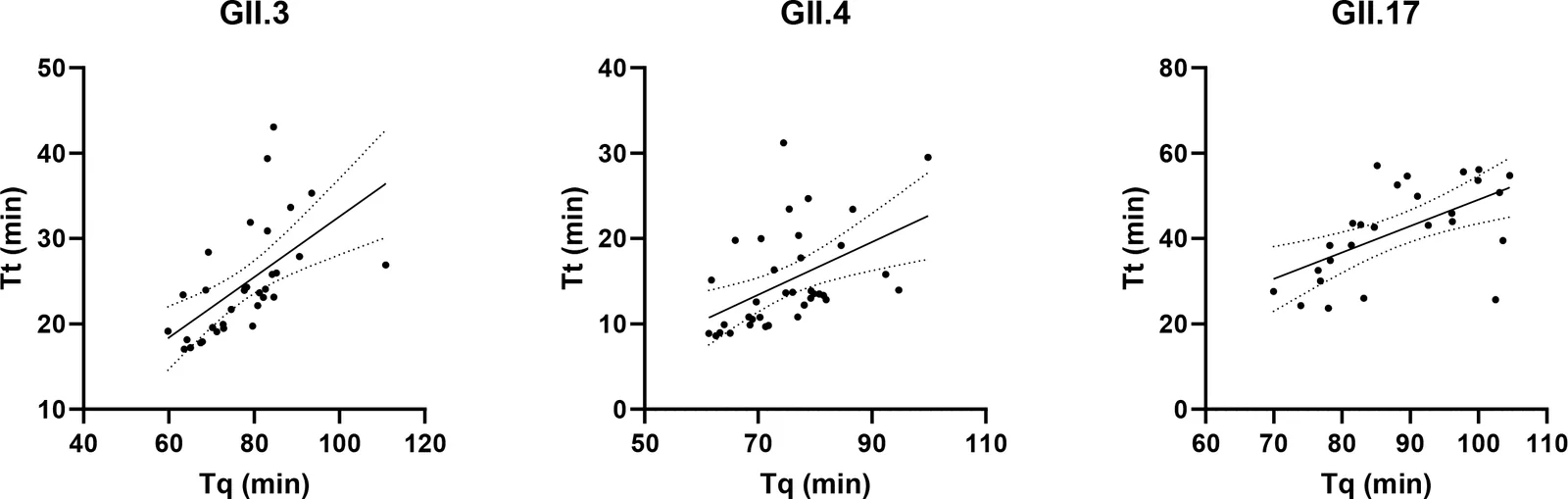
Correlation of threshold times between Lab-CPA and RT-qPCR. Scatter plots showing the linear correlation between Lab-CPA threshold time (Tt) and RT-qPCR equivalent time (Tq) for each genotype. Data points represent individual reactions. Solid lines indicate linear regression fits, and dashed lines represent 95% confidence intervals. Tq, the actual running time in RT-qPCR when the Ct value is reached; Tt, the time required for the fluorescence signal to cross the threshold in Lab-CPA.

### The Lab-CPA systems demonstrated successful integration with the EasyNAT platform

To enable future deployment, we adapted the Lab-CPA workflow for integration into the EasyNAT platform. As illustrated in Fig. 3A, this system uses proprietary all-in-one cartridges that perform nucleic acid extraction and CPA amplification in a single reaction chamber. We evaluated the performance of the integrated EasyNAT platform against the reference RT-qPCR assay using 84 fecal samples (24 GII.3-positive samples, 23 GII.4-positive samples, 19 GII.17-positive samples, and 18 norovirus-negative samples). The EasyNAT systems demonstrated adequate diagnostic accuracy across all three genotypes, showing PPAs of 91.7%, 95.7%, and 84.2%, NPAs of 100%, and TPAs of 95.2%, 97.6%, and 91.9% for GII.3, GII.4, and GII.17, respectively (Table 4). Kappa analysis confirmed substantial agreement with the reference standard (*κ* = 0.904, 0.951, and 0.838 for GII.3, GII.4, and GII.17, respectively).

**Fig 3:**
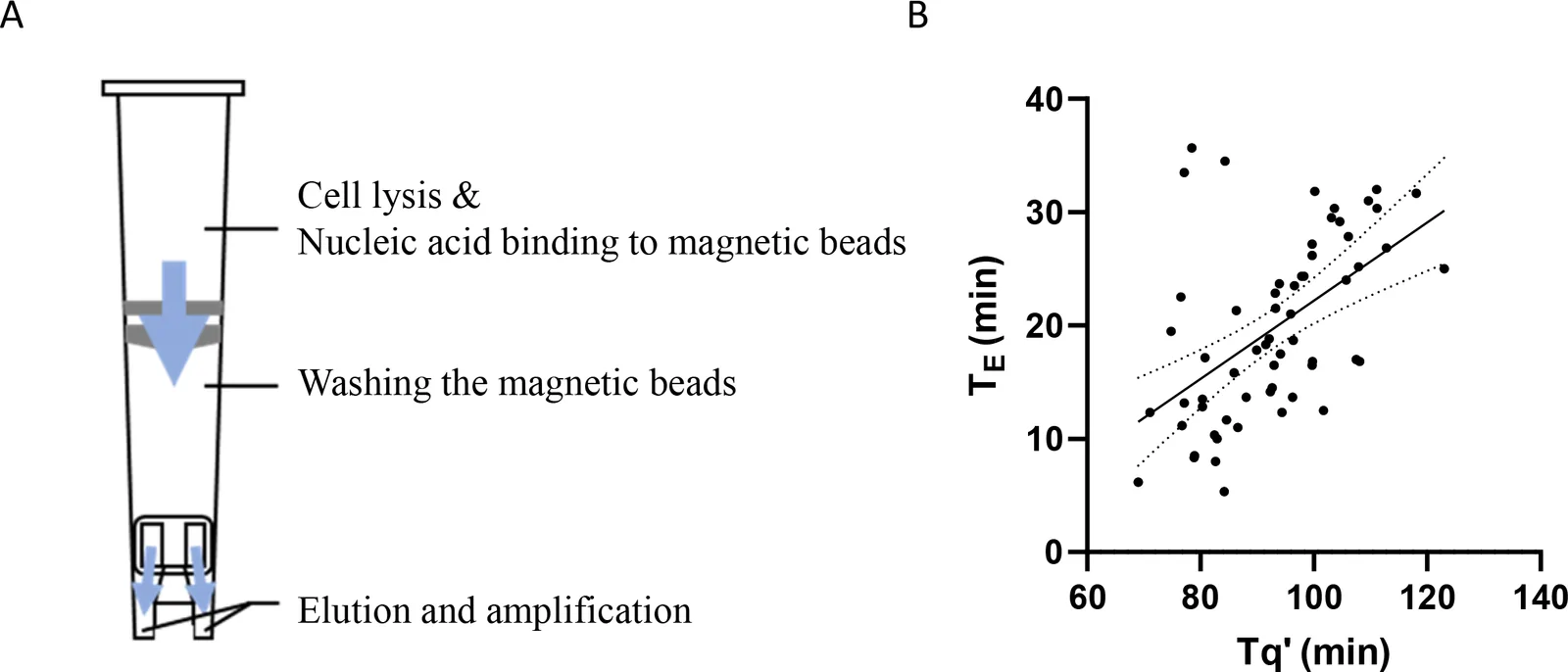
Integration of the CPA system with the EasyNAT platform. (**A**) Schematic workflow of the fully automated EasyNAT cartridge. The closed system integrates magnetic bead-based nucleic acid extraction (cell lysis and binding), automated washing steps, and CPA amplification in a single reaction chamber. (**B**) Scatter plot showing the linear correlation between the threshold time of EasyNAT (*T*_E_) and the equivalent time of RT-qPCR (Tq′). Data points represent individual samples, with the solid line indicating the linear regression fit and the dashed lines representing the 95% CI. T_E_, the total time from sample loading until the fluorescence signal crosses the threshold in the integrated cartridge. Tq’, the total time required for nucleic acid extraction and RT-qPCR amplification.

**TABLE 4:** Performance of the EasyNAT systems compared with RT-qPCR^*a*^

		RT-qPCR		PPA (%)	NPA (%)	TPA (%)
		Positive	Negative			
GII.3 EasyNAT	Positive	22	0	91.7	100.0	95.2
	Negative	2	18			
GII.4 EasyNAT	Positive	22	0	95.7	100.0	97.6
	Negative	1	18			
GII.17 EasyNAT	Positive	16	0	84.2	100.0	91.9
	Negative	3	18			

^*a*^ NPA, negative percent agreement; PPA, positive percent agreement; TPA, total percent agreement.

Next, we investigated the linear correlation of total running time between the two platforms. Considering that *T*_E_ represented the total time from sample input to positive signal detection for EasyNAT, encompassing both nucleic acid extraction and amplification, a new metric, termed Tq′, was introduced to represent the equivalent time for RT-qPCR assay. As depicted in Fig. 3B, a strong positive linear correlation was observed between *T*_E_ and Tq′ (*r* = 0.532, *P* < 0.0001). This finding indicates that the amplification dynamics of EasyNAT systems are closely synchronized with the RT-qPCR assay. Collectively, these data verify the successful adaptation of Lab-CPA to the commercial EasyNAT platform and highlight its great potential for rapid, fully automated clinical application.

## DISCUSSION

Drawing from clinical practice, norovirus infections predominantly manifest as sporadic cases rather than large-scale outbreaks, frequently presenting in outpatient clinics and emergency departments with acute gastroenteritis (5, 6). In these fast-paced environments, where timely clinical decision-making is critical, the conventional delay associated with RT-qPCR is often unacceptable. To address this, we introduced a novel detection method based on CPA technology, which significantly enhances operational efficiency while demonstrating substantial concordance with RT-qPCR. Additionally, the capability of our CPA systems to identify specific genotypes could enhance clinical management of norovirus infections (31, 32).

The current mainstream isothermal amplification methodologies, including LAMP (33–35) and RPA (23, 24, 36), possess inherent technical drawbacks that restrict their practical applications. For instance, LAMP has been reported to show unsatisfactory linearity in quantitative analyses (33). For RPA, its low reaction temperature is an important factor that possibly induces non-specific amplification (37, 38). Moreover, RPA is highly sensitive to template purity, and samples like feces—rich in inhibitors such as humic acids and bile salts—frequently compromise reaction efficiency (39, 40). In comparison, as a promising substitute in our country, CPA offers distinct advantages that possibly circumvent these challenges. As demonstrated in this study, CPA exhibited a significant positive linear correlation with the RT-qPCR assay, suggesting the potential for future quantitative applications. Also, CPA operates at a higher reaction temperature than RPA (~60°C vs ~40°C), which may inhibit non-specific amplification and enhance the tolerance to inhibitors in complex sample matrices (41–43). Moreover, the successful clinical implementation of the EasyNAT system across multiple pathogen detection scenarios substantiates its stability and reliability (21, 24, 25). Coupled with its significant cost advantage, CPA represents a more applicable and scalable solution for widespread adoption in our country.

It is noteworthy that the EasyNAT systems demonstrated lower PPAs and TPAs in comparison to the Lab-CPA systems in this study, a difference likely stemming from variations in nucleic acid extraction workflows. While both systems rely on magnetic bead-based extraction, Lab-CPA uses a standard standalone procedure, whereas EasyNAT operates via an integrated workflow within a sealed cartridge, which brings multiple technical limitations. A key factor may be that EasyNAT extracts relatively crude nucleic acids that are co-eluted with more impurities, including proteins, polysaccharides, and residual guanidine salts (24, 25). These contaminants exert inhibitory effects on amplification enzymes. In contrast, the conventional magnetic bead protocol of Lab-CPA incorporates rigorous washing steps to purify target nucleic acids and remove inhibitory substances efficiently, thereby yielding templates of superior quality. Additionally, it should be mentioned that the EasyNAT validation involved a smaller sample size than that of Lab-CPA in this study, which may amplify random variations and suboptimal assay performance. This factor, together with the technical limitations of the cartridges, collectively contributed to the slightly lower overall agreement of the EasyNAT systems.

Among all three genotypes of norovirus that were detected, the GII.17 systems demonstrated inferior overall performance in comparison to GII.3 and GII.4, with more pronounced declines in PPA and TPA when deployed on the EasyNAT system. In addition to the technical limitations of the EasyNAT cartridge platform previously mentioned, the intrinsic factors of primers, such as weak binding affinity, may also have the potential to make it more susceptible to interference from low viral loads and amplification inhibitors, thus compromising its performance. Additionally, the sample size used in the validation of GII.17 systems, both Lab-CPA and EasyNAT, is the smallest among the three genotypes, which may also contribute to the amplification of random variations, resulting in seemingly poorer performance. Hence, further refinement of primer design, amplification parameters, accompanied by systematic and large-scale validation, is promising to address these issues and promote the clinical application of the CPA technology for the detection of diverse norovirus genotypes.

This study has several limitations that warrant consideration. First, the current CPA system targets only the predominant GII.3, GII.4, and GII.17 genotypes, contrasting with the pan-genotypic detection capability of clinical RT-qPCR kits. This constraint arises from the inherent challenge of identifying conserved genomic regions across norovirus’s highly divergent genotypes suitable for CPA. Nevertheless, the EasyNAT system enables the incorporation of different primer sets targeting distinct genotypes into a single reaction cartridge. Specific genotypes can be identified through distinct fluorescent reporter colors and separate reaction chambers within the cartridge, allowing for the future integration of multiplex primer panels to expand genotype coverage. Second, while all specimens were maintained at −80°C under standardized protocols, variations in testing timelines may have introduced RNA degradation artifacts, potentially impacting viral load stability and assay reproducibility. Third, while the eight-sample throughput suits decentralized testing, high-volume laboratories may require parallel analyzer deployment. Nevertheless, the system’s portability and rapid turnaround effectively balance throughput constraints against POCT requirements, making it a practical choice for a wide range of applications.

This study establishes CPA technology as a transformative solution for norovirus diagnostics. By achieving a high level of agreement with RT-qPCR while significantly reducing processing time, our CPA systems demonstrate the viability for both hospital-based testing and resource-limited settings. This dual capability expands the scope of molecular diagnostics, enabling the rapid, on-site identification of emerging and re-emerging pathogens beyond traditional laboratories.

## Supplementary Material

Reviewer comments

## Data Availability

The data sets analyzed during the current study are available from the corresponding author on reasonable request.

## SUPPLEMENTAL MATERIAL

Fig. S1; Table S1 spectrum.01684-26-s0001.pdf Table S1: Reaction components of the Lab-CPA system. Fig. S1: Phylogenetic trees of norovirus strains.

OPEN PEER REVIEW reviewer-comments.pdf An accounting of the reviewer comments and feedback.

ASM does not own the copyrights to Supplemental Material that may be linked to, or accessed through, an article. The authors have granted ASM a non-exclusive, world-wide license to publish the Supplemental Material files. Please contact the corresponding author directly for reuse.
