# Supplementary material for: Isothermal cross-priming amplification for field-deployable norovirus surveillance: a cost-effective and rapid solution
Source: Microbiol Spectr. 2026 Sep 4;14(10):e01684-26. doi: 10.1128/spectrum.01684-26 (PMC13689371; doi:10.1128/spectrum.01684-26)
Supplement: Supplementary file 1 [file reviewer-comments.pdf]

ASM | Microbiology Spectrum

Isothermal Cross-Priming Amplification for Field-Deployable Norovirus Surveillance: A Cost-Effective and Rapid Solution

Ran Jia, Lijuan lu, Xunhua Zhu, Lingfeng Cao, Liyun Su, Huaqing Zhong, Menghua Xu, pengcheng liu, Yanqiong Zhou, and Jin Xu

Corresponding Author(s): Jin Xu, Department of Clinical Laboratory, Children's Hospital of Fudan University

Review Timeline:

Submission Date:	May 15, 2026
Editorial Decision:	June 2, 2026
Revision Received:	June 26, 2026
Editorial Decision:	August 4, 2026
Revision Received:	August 7, 2026
Accepted:	August 7, 2026

Editor: Gabriel Parra

Reviewer(s): The reviewers have opted to remain anonymous.

Transaction Report:

(Note: With the exception of the correction of typographical or spelling errors that could be a source of ambiguity, letters and reports are not edited. The original formatting of letters and referee reports may not be reflected in this compilation.)

DOI: https://doi.org/10.1128/spectrum.01684-26

Re: Spectrum01684-26 (**Isothermal Cross-Priming Amplification for Field-Deployable Norovirus Surveillance: A Cost-Effective and Rapid Solution**)

Dear Dr. Jin Xu:

Thank you for submitting your manuscript to Microbiology Spectrum. Two reviewers evaluated your manuscript and, while they found the subject matter to be of interest, both recommended revisions.

Please note that additional detail should be provided in the Methods section to ensure that (reviewers and) readers can fully understand the assays described and, where applicable, reproduce the experiments. Clear and comprehensive methodological descriptions are essential for the evaluation and interpretation of the study.

Please return the manuscript within 60 days; if you cannot complete the modification within this time period, please contact me. If you do not wish to modify the manuscript and prefer to submit it to another journal, notify me immediately so that the manuscript may be formally withdrawn from consideration by Spectrum.

Revision Guidelines
To submit your modified manuscript, log into the submission site at https://spectrum.msubmit.net/cgi-bin/main.plex. Go to Author Tasks and click the appropriate manuscript title to begin. The information you entered when you first submitted the paper will be displayed; update this as necessary. Note the following requirements:

• Upload point-by-point responses to the issues raised by the reviewers in a file named "Response to Reviewers," NOT in your cover letter.
• Upload a compare copy of the manuscript (without figures) as a "Marked-Up Manuscript" file.
• Upload a clean .DOC/.DOCX version of the revised manuscript and remove the previous version.
• Each figure must be uploaded as a separate, editable, high-resolution file (TIFF or EPS preferred), and any multipanel figures must be assembled into one file.
• Any supplemental material intended for posting by ASM should be uploaded with their legends separate from the main manuscript. You can combine all supplemental material into one file (preferred) or split it into a maximum of 10 files with all associated legends included.

For complete guidelines on revision requirements, see our Submission and Review Process webpage. Submission of a paper that does not conform to guidelines may delay acceptance of your manuscript.

Data availability: ASM policy requires that data be available to the public upon online posting of the article, so please verify all links to sequence records, if present, and make sure that each number retrieves the full record of the data. If a new accession number is not linked or a link is broken, provide Spectrum production staff with the correct URL for the record. If the accession numbers for new data are not publicly accessible before the expected online posting of the article, publication may be delayed; please contact production staff (Spectrum@asmusa.org) immediately with the expected release date.

Publication Fees: For information on publication fees and which article types are subject to charges, visit our website. If your manuscript is accepted for publication and any fees apply, you will be contacted separately about payment during the production process; please follow the instructions in that e-mail. Arrangements for payment must be made before your article is published.

ASM Membership: Corresponding authors may join or renew ASM membership to obtain discounts on publication fees. Need to upgrade your membership level? Please contact Customer Service at Service@asmusa.org.

The ASM Journals program strives for constant improvement in our submission and publication process. Please tell us how we can improve your experience by taking this quick Author Survey.

Thank you for submitting your paper to Spectrum.

Sincerely,
Gabriel Parra
Editor
Microbiology Spectrum

Reviewer #1 (Comments for the Author):

In this manuscript, the authors describe the establishment of a cross-priming amplification (Lab-CPA) system targeting predominant norovirus genotypes in China: GII.3, GII.4, and GII.17. This system achieved high sensitivity and specificity and

reduced the processing time compared to conventional RT-PCR methods. This technology is useful for norovirus surveillance and the response to outbreaks in China.

Minor Comments:

1. Please provide the IRB study number in the methods.

2. Page 7: Please briefly describe the phylogenetic tree (Sup Fig 1) in the text. Just stating that "the phylogenetic tree is also displayed" is not sufficient.

3. The formatting for Table 2 is confusing. I recommend dividing the Results column into two sub-columns: positive and negative. Thus, the actual numbers of positive and negative samples could be reported in these sub-columns, similar to Tables 3 and 4.

4. Is there an explanation as to why the PPAs are lower for the Easy-NAT system vs RT-PCR compared to the Lab-CPA system vs RT-PCR?

5. Page 10: Please define "POCT" in the text.

6. Page 4: Please modify text to change the period after citation [12] to a comma: "...while GII.4 circulates broadly among pediatric populations, causing typical gastroenteritis [12], GII.17 preferentially targets older children and is associated with severe, projectile vomiting and heightened dehydration risk [8, 10]."

Reviewer #2 (Comments for the Author):

The writing of the paper is unclear and incoherent. The methods and results are poorly described and the discussion is incomplete and incoherent. All of these makes the evaluation of the paper very difficult if not impossible.
Introduction:
• The introduction is short and rather incomplete. The LAMP and CPA systems should be explained and their differences with conventional RT-PCR should be described. The aim of the study should be clearly justified.
• The EasyNAT system needs explanation in the introduction section.
Methods:
• How were the primers designed?
• How was the phylogenetic tree constructed?
• How was the specificity and sensitivity of the assay assessed?
• There is no description of the biosafety procedures.
Results & discussion:
• There is no justification for the genotypes that are chosen for this study.
• Table 2 is unclear and the presentation of data is not appropriate.
• Figure 2 and 3 are not adequately described.
• What is kappa analysis?
• Why the assay performed poorly for GII.17 samples?
• There are universal primers for NoV GI and GII, that allow detection of norovirus irrespective of genotype, while this assay requires specific primers for each genotype. Therefore it is not applicable in the system as first it is needed to genotype the samples to figure out which primers to use for the assay. This makes the assay unpractical.

In this manuscript, the authors describe the establishment of a cross-priming amplification (Lab-CPA) system targeting predominant norovirus genotypes in China: GII.3, GII.4, and GII.17. This system achieved high sensitivity and specificity and reduced the processing time compared to conventional RT-PCR methods. This technology is useful for norovirus surveillance and the response to outbreaks in China.

Minor Comments:

1. Please provide the IRB study number in the methods.

2. Page 7: Please briefly describe the phylogenetic tree (Sup Fig 1) in the text. Just stating that "the phylogenetic tree is also displayed" is not sufficient.

3. The formatting for Table 2 is confusing. I recommend dividing the Results column into two sub-columns: positive and negative. Thus, the actual numbers of positive and negative samples could be reported in these sub-columns, similar to Tables 3 and 4.

4. Is there an explanation as to why the PPAs are lower for the Easy-NAT system vs RT-PCR compared to the Lab-CPA system vs RT-PCR?

5. Page 10: Please define "POCT" in the text.

6. Page 4: Please modify text to change the period after citation [12] to a comma: "...while GII.4 circulates broadly among pediatric populations, causing typical gastroenteritis [12], GII.17 preferentially targets older children and is associated with severe, projectile vomiting and heightened dehydration risk [8, 10].

Point-by-point responses

Reviewer #1 (Comments for the Author):

In this manuscript, the authors describe the establishment of a cross-priming amplification (Lab-CPA) system targeting predominant norovirus genotypes in China: GII.3, GII.4, and GII.17. This system achieved high sensitivity and specificity and reduced the processing time compared to conventional RT-PCR methods. This technology is useful for norovirus surveillance and the response to outbreaks in China.

Response: We appreciate your insightful comments and have revised the text in accordance with your valuable recommendations.

Minor Comments:

Comment 1. Please provide the IRB study number in the methods.

Response: We are grateful for your thoughtful notification and have incorporated the IRB number as indicated below (see **Page 7, lines 187-188**).

"The study was approved by the Ethics Committee of Children's Hospital of Fudan University [NO. 2025(434)]."

Comment 2. Page 7: Please briefly describe the phylogenetic tree (Sup Fig 1) in the text. Just stating that "the phylogenetic tree is also displayed" is not sufficient.

Response: We sincerely apologize for the oversight, and have supplemented the relevant details in the Materials and methods section (see **Page 8, lines 209-217**) and the Results section (see **Page 10, lines 274-279**), as outlined below.

In the Materials and methods section:

"Phylogenetic Analysis

Our CPA detection systems focused specifically on the GII.3, GII.4, and GII.17 genotypes due to their epidemiological predominance [6]. Representative strains of the three genotypes were chosen for phylogenetic reconstruction. Using the MUSCLE algorithm, the nucleotide sequences of the VP1 region derived from this study were aligned with reference sequences obtained from GenBank. Phylogenetic trees were constructed using the neighbor-joining method, and branch support was assessed using 1,000 bootstrap iterations. All phylogenetic analyses were performed using MEGA software (version 11)."

In the Result section:

"A phylogenetic tree was constructed based on sequence alignments of the target strains and reference sequences retrieved from GenBank (Supplementary Figure 1). The resulting topology revealed that the viral strains selected for validation are phylogenetically distinct and segregate into the three targeted genotypes—GII.3, GII.4, and GII.17—thereby confirming the accuracy of our genotyping result."

Comment 3. The formatting for Table 2 is confusing. I recommend dividing the Results column into

two sub-columns: positive and negative. Thus, the actual numbers of positive and negative samples could be reported in these sub-columns, similar to Tables 3 and 4.

Response: We would like to apologize for our confusing formatting. As recommended, Table 2 has been modified to include two sub-columns in the results column, with important metrics such as PPV, NPV, sensitivity, and specificity being added. The description of Table 2 has also been revised as outlined below (see **Page 11, lines 280-291**).

"For the validation of the GII.3 Lab-CPA system, a total of 102 samples were tested. It correctly identified 49 of 50 GII.3-positive samples and all 52 GII.3-negative samples, yielding a positive predictive value (PPV) of 100.0% (49/49), and a negative predictive value (NPV) of 98.1% (52/53), a sensitivity of 98.0% (49/50), and a specificity of 100.0% (52/52) (Table 2). The GII.4 Lab-CPA system demonstrated excellent performance across all 164 samples (80 GII.4-positive, 84 GII.4-negative), achieving 100% for all diagnostic metrics (Table 2). The GII.17 Lab-CPA system was evaluated using 80 samples, identifying 31 of 32 positive cases and all 48 negative controls. This resulted in a PPV of 100.0% (31/31), an NPV of 98.0% (48/49), a sensitivity of 96.9% (31/32), and a specificity of 100.0% (48/48) (Table 2). Collectively, these data confirm that the three Lab-CPA systems exhibit robust and reliable performance for detecting NoV GII.3, GII.4, and GII.17."

Comment 4. Is there an explanation as to why the PPAs are lower for the Easy-NAT system vs RT-PCR compared to the Lab-CPA system vs RT-PCR?

Response: We greatly appreciate your thought-provoking inquiry. A new paragraph has been incorporated into the Discussion section in order to provide an explanation of the potential underlying reasons (see **Page 14, lines 371-386**).

"It is noteworthy that the EasyNAT systems demonstrated lower PPAs and TPAs in comparison to the Lab-CPA systems in this study, a difference likely stemming from variations in nucleic acid extraction workflows. While both systems rely on magnetic bead-based extraction, Lab-CPA uses a standard standalone procedure, whereas EasyNAT operates via an integrated workflow within a sealed cartridge, which brings multiple technical limitations. A key factor may be that EasyNAT extracts relatively crude nucleic acids that are co-eluted with more impurities, including proteins, polysaccharides, and residual guanidine salts [24, 25]. These contaminants exert inhibitory effects on amplification enzymes. In contrast, the conventional magnetic bead protocol of Lab-CPA incorporates rigorous washing steps to purify target nucleic acids and remove inhibitory substances efficiently, thereby yielding templates of superior quality. Additionally, it should be mentioned that the EasyNAT validation involved a smaller sample size than that of Lab-CPA in this study, which may amplify random variations and suboptimal assay performance. This factor, together with the technical limitations of the cartridges, collectively contributed to the slightly lower overall agreement of the EasyNAT systems."

Comment 5. Page 10: Please define "POCT" in the text.

Response: Please accept our apologies for the omission. The term "POCT" has now been defined in the revised manuscript as follows (see **Page 6, lines 155-158**).

"This technology has been fully integrated into the commercially available EasyNAT

molecular diagnostic platform [21-23], which is designed for point-of-care testing (POCT). Characterized by rapid on-site detection of patient samples, POCT requires portable analysis and immediate result readout [22]. ”

Comment 6. Page 4: Please modify text to change the period after citation [12] to a comma: "...while GII.4 circulates broadly among pediatric populations, causing typical gastroenteritis [12], GII.17 preferentially targets older children and is associated with severe, projectile vomiting and heightened dehydration risk [8, 10]."
Response: We greatly apologize for the mistake. The correction has been made to **Page 5, line 131**.

“While GII.4 circulates broadly across all age groups, causing typical gastroenteritis [13], GII.17 preferentially targets older children and is associated with severe, projectile vomiting and heightened dehydration risk [9, 11, 14].”

Reviewer #2 (Comments for the Author):

The writing of the paper is unclear and incoherent. The methods and results are poorly described and the discussion is incomplete and incoherent. All of these makes the evaluation of the paper very difficult if not impossible.
Response: We would like to express our sincerest apologies for the unsatisfactory quality of our writing. We have carefully made the modifications, corrections, and supplements to this manuscript in alignment with your valuable recommendations, with the objective of enhancing the overall readability of the text.

Introduction:
Comment 1. The introduction is short and rather incomplete. The LAMP and CPA systems should be explained and their differences with conventional RT-PCR should be described. The aim of the study should be clearly justified.
Response: We are grateful for your kind suggestions. The introduction has undergone modification and supplementation with a description of LAMP, RPA, CPA (EasyNAT), as well as their comparison with RT-qPCR (see **Page 5-7, lines 137-167**). Also, we have modified our description of the aim of the study as below (see **Page 7, lines 168-170**).

Description of LAMP, RPA, CPA (EasyNAT):

“Current diagnostic procedures for NoV infection rely heavily on reverse transcription quantitative PCR (RT-qPCR). However, the typical workflow of this technique requires multiple steps, including nucleic acid extraction, reverse transcription, and real-time PCR cycling, which is time-consuming (~2–3 h) and lacks portability [17]. To address these limitations, several isothermal amplification technologies have emerged as promising alternatives, including loop-mediated isothermal amplification (LAMP) and recombinase polymerase amplification (RPA) [18-20]. As reported, LAMP is capable of achieving 10^9-10^{10}-fold amplification within 15–60 minutes under constant temperature (~65°C) [18, 19], and has been shown to yield detection rates that are comparable to RT-qPCR for specific pathogens [18]. RPA is also a highly specific and sensitive isothermal amplification technique (~40°C), and it has been successfully adopted for the detection

of various target pathogens [20]. However, these technologies are protected by foreign patents, resulting in higher costs and deployment constraints in our country.

Cross-priming amplification (CPA) is a domestically patented isothermal technology that has the potential to substitute LAMP and RPA in our country. This technology utilizes Bst DNA polymerase with strand displacement specificity and betaine to efficiently amplify the target sequence at a constant temperature (~63 °C) within 40-60 minutes [21]. This technology has been fully integrated into the commercially available EasyNAT molecular diagnostic platform [21-23], which is designed for point-of-care testing (POCT). Characterized by rapid on-site detection of patient samples, POCT requires portable analysis and immediate result readout [22]. To meet these demands, the EasyNAT platform seamlessly combines magnetic bead-based nucleic acid extraction and CPA amplification within a single reaction chamber, thereby minimizing hands-on time and reducing contamination risk [24]. So far, it has demonstrated strong clinical performance in comparison to RT-qPCR across multiple targets, including Mycobacterium tuberculosis, SARS-CoV-2, Chlamydia trachomatis/Neisseria gonorrhoeae, and Mycoplasma pneumonia [21, 24, 25]. Hence, the combination of operational simplicity, minimal infrastructure requirements, adequate accuracy, and rapid processing time positions EasyNAT as a practical alternative to lab-bound RT-qPCR assays for frontline screening and near-patient diagnosis. ”

Aim of the study:

“Building on the untapped potential of CPA technology for NoV testing, this study aimed to develop and validate a novel CPA assay enabling both rapid detection and concurrent genotyping.”

Comment 2. The EasyNAT system needs explanation in the introduction section.

Response: We are grateful for your kind suggestion. We have added the description of EasyNAT in the Introduction (see **Page 6, lines 154-167**, please refer to the response to Comment 1). Also, we have supplemented details about EasyNAT cartridges in the Materials and methods section as below (see **Page 9, lines 238-245**).

“CPA assay based on the EasyNAT detection system

The Lab-CPA systems were integrated into the EasyNAT platform, utilizing a fully automated cartridge that consolidates magnetic bead-based nucleic acid extraction and CPA amplification. The cartridge architecture features a main chamber and two dependent side chambers pre-loaded with genotype-specific CPA primers/probes, Bst DNA polymerase, and reaction buffer (Figure 3C). Upon loading the fecal sample, the EasyNAT analyzer executes a predefined thermal-kinetic program to drive the integrated workflow. Amplification kinetics are monitored in real time, yielding a time-to-threshold value (T_E) for quantitative analysis.”

Comment 3. How were the primers designed?

Response: We greatly apologize for omitting the information. The design of the primers has been described as follows (see **Page 8-9, lines 220-229**).

“Primers/probes were designed based on the specific principles of CPA, which requires a set of 6 primers/probes to initiate strand displacement and exponential amplification [27]. The VP1 gene was specifically targeted, as it contains conserved regions suitable for genotyping. To further

identify the conserved motifs, we aligned multiple reference sequences of the GII.3, GII.4, and GII.17 genotypes obtained from GenBank. Based on these alignments, genotype-specific primer sets were designed using SnapGene software (version 2.3.2) (Table 1 and Supplementary Table 1). Great care was taken to ensure optimal melting temperatures and to avoid secondary structures that could inhibit the isothermal reaction. The primers/probes were synthesized by Sangon Biotech (Shanghai, China)."

Comment 4. How was the phylogenetic tree constructed?

Response: Please accept our sincere apologies for the oversight. We have supplemented the relevant details in the Materials and methods section (see **Page 8, lines 209-217**) and the Result section (see **Page 10, lines 274-279**), as outlined below.

In the Method section:

"Phylogenetic Analysis

Our CPA detection systems focused specifically on the GII.3, GII.4, and GII.17 genotypes due to their epidemiological predominance [6]. Representative strains of the three genotypes were chosen for phylogenetic reconstruction. Using the MUSCLE algorithm, the nucleotide sequences of the VP1 region derived from this study were aligned with reference sequences obtained from GenBank. Phylogenetic trees were constructed using the neighbor-joining method, and branch support was assessed using 1,000 bootstrap iterations. All phylogenetic analyses were performed using MEGA software (version 11)."

In the Result section

"A phylogenetic tree was constructed based on sequence alignments of the target strains and reference sequences retrieved from GenBank (Supplementary Figure 1). The resulting topology revealed that the viral strains selected for validation are phylogenetically distinct and segregate into the three targeted genotypes—GII.3, GII.4, and GII.17—thereby confirming the accuracy of our genotyping result."

Comment 5. How was the specificity and sensitivity of the assay assessed?

Response: We apologize for our confusing formatting. We have modified Table 2 and added important metrics such as PPV, NPV, sensitivity, and specificity. The description of Table 2 has also been revised as outlined below (see **Page 11, lines 280-291**).

"For the validation of the GII.3 Lab-CPA system, a total of 102 samples were tested. It correctly identified 49 of 50 GII.3-positive samples and all 52 GII.3-negative samples, yielding a positive predictive value (PPV) of 100.0% (49/49), and a negative predictive value (NPV) of 98.1% (52/53), a sensitivity of 98.0% (49/50), and a specificity of 100.0% (52/52) (Table 2). The GII.4 Lab-CPA system demonstrated excellent performance across all 164 samples (80 GII.4-positive, 84 GII.4-negative), achieving 100% for all diagnostic metrics (Table 2). The GII.17 Lab-CPA system was evaluated using 80 samples, identifying 31 of 32 positive cases and all 48 negative controls. This resulted in a PPV of 100.0% (31/31), an NPV of 98.0% (48/49), a sensitivity of 96.9% (31/32), and a specificity of 100.0% (48/48) (Table 2). Collectively, these data confirm that the three Lab-CPA systems exhibit robust and reliable performance for detecting NoV GII.3, GII.4, and GII.17."

Comment 6. There is no description of the biosafety procedures.

Response: We deeply apologize for the oversight and have included the descriptions of the biosafety procedures in the Materials and methods section as below (see **Page 7, lines 182-188**).

"All of the clinical specimen processing and detection was performed within the accredited biosafety level 2 (BSL-2) laboratory at the Clinical Laboratory Center, Children's Hospital of Fudan University. All operations were conducted in class II biosafety cabinets with standardized personal protective equipment. All biohazardous waste was autoclaved (121°C, 30 min) before disposal, and surfaces were routinely decontaminated following institutional biosafety protocols. The study was approved by the Ethics Committee of Children's Hospital of Fudan University [NO. 2025(434)]."

Results & discussion:

Comment 7. There is no justification for the genotypes that are chosen for this study.

Response: We apologize for the unclear explanation regarding the selection of genotypes. To clarify, we have included additional details about the dominant genotypes in the Introduction section to offer a stronger epidemiological rationale (see **Page 5, lines 123-129**). Additionally, we have referenced our recent epidemiological study [**New Ref 6**], which identified the main genotypes found in children with sporadic acute gastroenteritis in Shanghai, China. This further supports our decision to concentrate on the three specific genotypes to ensure the clinical relevance and practical application of our assay.

"In China, GII.4 and GII.3 have historically dominated, accounting for over 90% of outbreaks and sporadic cases in China in the past decade [4, 5]. However, recent surveillance, including our own epidemiological investigation [6], indicates a significant shift, with emergent GII.17 strains superseding GII.4 as the predominant genotype across Europe [7-9], the United States [10, 11], and China [12]. Therefore, to ensure broad coverage and clinical utility, this study specifically targeted the three most prevalent genotypes: GII.3, GII.4, and GII.17."

The new reference:

6. Lu LJ, Zhong HQ, Jia R, Xu MH, Liu PC, Zhu XH, Xu J: Resurgence and Predominance of GII.17[P17] Norovirus in Children With Sporadic Acute Gastroenteritis in Shanghai, China. J Med Virol 2025, 97:e70729.

Comment 8. Table 2 is unclear and the presentation of data is not appropriate.

Response: Thank you for the constructive suggestion. Table 2 has been modified and supplemented with important metrics. Also, we have added essential descriptions on these metrics as below (see **Page 10-11, lines 278-289**).

"For the validation of the GII.3 Lab-CPA system, a total of 102 samples were tested. It correctly identified 49 of 50 GII.3-positive samples and all 52 GII.3-negative samples, yielding a positive predictive value (PPV) of 100.0% (49/49), and a negative predictive value (NPV) of 98.1% (52/53), a sensitivity of 98.0% (49/50), and a specificity of 100.0% (52/52) (Table 2). The GII.4 Lab-CPA system demonstrated excellent performance across all 164 samples (80 GII.4-positive, 84

GII.4-negative), achieving 100% for all diagnostic metrics (Table 2). The GII.17 Lab-CPA system was evaluated using 80 samples, identifying 31 of 32 positive cases and all 48 negative controls. This resulted in a PPV of 100.0% (31/31), an NPV of 98.0% (48/49), a sensitivity of 96.9% (31/32), and a specificity of 100.0% (48/48) (Table 2). Collectively, these data confirm that the three Lab-CPA systems exhibit robust and reliable performance for detecting NoV GII.3, GII.4, and GII.17."

Comment 9. Figure 2 and 3 are not adequately described.

Response: We extend our sincere apologies for any confusion caused by the unclear writing. Amendments have been made to the descriptions of Figures 2 and 3, both in the Results section and in the figure legends (see **Page 11-13, lines 306-314, 323-342**).

Figure 2:

"To further investigate the consistency between the Lab-CPA systems and the RT-qPCR assay, we analyzed the correlation of their threshold times. Given that the Lab-CPA systems could output a threshold time (Tt) directly, the RT-qPCR cycle threshold (Ct) values were converted into equivalent reaction times (Tq) on the basis of the thermal cycling parameters. As a result, a significant positive linear correlation was observed between Tt and Tq for all three genotypes (GII.3: $r = 0.584$, $p = 0.0004$; GII.4: $r = 0.487$, $p = 0.0026$; GII.17: $r = 0.586$, $p = 0.0016$; **Figure 2**). This correlation suggests that the amplification kinetics of the Lab-CPA systems closely parallel those of the reference RT-qPCR method."

Figure 3:

"To enable future deployment, we adapted the Lab-CPA workflow for integration into the EasyNAT platform. As illustrated in **Figure 3A**, this system uses proprietary all-in-one cartridges that perform nucleic acid extraction and CPA amplification in a single reaction chamber. We evaluated the performance of the integrated EasyNAT platform against the reference RT-qPCR assay using 84 fecal samples (24 GII.3-positive samples, 23 GII.4-positive samples, 19 GII.17-positive samples, and 18 NoV-negative samples). The EasyNAT systems demonstrated adequate diagnostic accuracy across all three genotypes, showing PPAs of 91.7%, 95.7%, and 84.2%, NPAs of 100%, and TPAs of 95.2%, 97.6%, and 91.9% for GII.3, GII.4, and GII.17, respectively (Table 4). Kappa analysis confirmed substantial agreement with the reference standard ($\kappa = 0.904$, 0.951, and 0.838 for GII.3, GII.4, and GII.17, respectively).

Next, we investigated the linear correlation of total running time between the two platforms. Considering that TE represented the total time from sample input to positive signal detection for EasyNAT, encompassing both nucleic acid extraction and amplification, a new metric, termed Tq', was introduced to represent the equivalent time for RT-qPCR assay. As depicted in **Figure 3B**, a strong positive linear correlation was observed between TE and Tq' ($r = 0.532$, $p < 0.0001$). This finding indicates that the amplification dynamics of EasyNAT systems are closely synchronized with the RT-qPCR assay. Collectively, these data verify the successful adaptation of Lab-CPA to the commercial EasyNAT platform and highlight its great potential for rapid, fully automated clinical application."

Comment 10. What is kappa analysis?

Response: We deeply apologize for the omission and have added the description of kappa analysis

in the Statistical method section as below (see **Page 10, lines 258-265**).

"To evaluate the agreement between two methods, we used Cohen's kappa coefficient (κ). Cohen's kappa measures the degree of agreement between two raters classifying items into mutually exclusive categories, while correcting for agreement that would be expected by chance alone [28, 29]. The kappa coefficient was interpreted according to the criteria proposed by Landis and Koch [30]: ≤ 0.20 indicates slight agreement; 0.21 - 0.40, fair; 0.41 - 0.60, moderate; 0.61 - 0.80, substantial; and 0.81 - 1.00, almost perfect agreement. All kappa analyses were performed using IBM SPSS Statistics software (version 23)."

Comment 11. Why the assay performed poorly for GII.17 samples?

Response: We appreciate your insightful question. The explanations about this may include primer-template kinetics, technical limitations in nucleic acid extraction, and sample size. We have explained these points in the Discussion section, with one paragraph addressing the technical limitations of EasyNAT and another analyzing the poorer performance specifically for GII.17 (see **Page 14-15, lines 371-399**).

"It is noteworthy that the EasyNAT systems demonstrated lower PPAs and TPAs in comparison to the Lab-CPA systems in this study, a difference likely stemming from variations in nucleic acid extraction workflows. While both systems rely on magnetic bead-based extraction, Lab-CPA uses a standard standalone procedure, whereas EasyNAT operates via an integrated workflow within a sealed cartridge, which brings multiple technical limitations. A key factor may be that EasyNAT extracts relatively crude nucleic acids that are co-eluted with more impurities, including proteins, polysaccharides, and residual guanidine salts [24, 25]. These contaminants exert inhibitory effects on amplification enzymes. In contrast, the conventional magnetic bead protocol of Lab-CPA incorporates rigorous washing steps to purify target nucleic acids and remove inhibitory substances efficiently, thereby yielding templates of superior quality. Additionally, it should be mentioned that the EasyNAT validation involved a smaller sample size than that of Lab-CPA in this study, which may amplify random variations and suboptimal assay performance. This factor, together with the technical limitations of the cartridges, collectively contributed to the slightly lower overall agreement of the EasyNAT systems.

Among all three genotypes of NoV that were detected, the GII.17 systems demonstrated inferior overall performance in comparison to GII.3 and GII.4, with more pronounced declines in PPA and TPA when deployed on the EasyNAT system. In addition to the technical limitations of the EasyNAT cartridge platform previously mentioned, the intrinsic factors of primers, such as weak binding affinity, may also have the potential to make it more susceptible to interference from low viral loads and amplification inhibitors, thus compromising its performance. Additionally, the sample size used in the validation of GII.17 systems, both Lab-CPA and EasyNAT, is the smallest among the three genotypes, which may also contribute to the amplification of random variations, resulting in seemingly poorer performance. Hence, further refinement of primer design, amplification parameters, accompanied by systematic and large-scale validation, is promising to address these issues and promote the clinical application of the CPA technology for the detection of diverse NoV genotypes."

Comment 12. There are universal primers for NoV GI and GII, that allow detection of norovirus irrespective of genotype, while this assay requires specific primers for each genotype. Therefore it is not applicable in the system as first it is needed to genotype the samples to figure out which primers to use for the assay. This makes the assay unpractical.

Response: We appreciate your valuable feedback. Our CPA systems do require specific primers for each genotype. However, the EasyNAT system allows the inclusion of primer sets targeting different genotypes within a single reaction cartridge. Specific genotypes can be identified using distinct fluorescent reporter colors and separate reaction chambers within the cartridge. Therefore, there is no need to genotype the samples beforehand; we can simultaneously detect GII.3, GII.4, and GII.17 directly from clinical samples without prior knowledge of the genotype.

We would like to express our sincere apologies for any confusion caused by our previous description and have added additional details to clarify this in the discussion section, as shown below (see **Page 15, lines 404-409**).

"Nevertheless, the EasyNAT system enables the incorporation of different primer sets targeting distinct genotypes into a single reaction cartridge, and specific genotypes can be identified through distinct fluorescent reporter colors and separate reaction chambers within the cartridge, allowing for the future integration of multiplex primer panels to expand genotype coverage."

Re: Spectrum01684-26R1 (**Isothermal Cross-Priming Amplification for Field-Deployable Norovirus Surveillance: A Cost-Effective and Rapid Solution**)

Dear Dr. Jin Xu:

Thank you for the privilege of reviewing your work. Your manuscript has improved significantly since the initial submission, and we are pleased to inform you that it is being considered for publication. Before we can proceed, however, we ask that you address the following minor comments and revisions. Below you will find my comments, instructions from the Spectrum editorial office, and the reviewer comments.

Please return the manuscript within 60 days; if you cannot complete the modification within this time period, please contact me. If you do not wish to modify the manuscript and prefer to submit it to another journal, notify me immediately so that the manuscript may be formally withdrawn from consideration by Spectrum.

Revision Guidelines
To submit your modified manuscript, log into the submission site at https://spectrum.msubmit.net/cgi-bin/main.plex. Go to Author Tasks and click the appropriate manuscript title to begin. The information you entered when you first submitted the paper will be displayed; update this as necessary. Note the following requirements:

• Upload point-by-point responses to the issues raised by the reviewers in a file named "Response to Reviewers," NOT in your cover letter.
• Upload a compare copy of the manuscript (without figures) as a "Marked-Up Manuscript" file.
• Upload a clean .DOC/.DOCX version of the revised manuscript and remove the previous version.
• Each figure must be uploaded as a separate, editable, high-resolution file (TIFF or EPS preferred), and any multipanel figures must be assembled into one file.
• Any supplemental material intended for posting by ASM should be uploaded with their legends separate from the main manuscript. You can combine all supplemental material into one file (preferred) or split it into a maximum of 10 files with all associated legends included.

For complete guidelines on revision requirements, see our Submission and Review Process webpage. Submission of a paper that does not conform to guidelines may delay acceptance of your manuscript.

Data availability: ASM policy requires that data be available to the public upon online posting of the article, so please verify all links to sequence records, if present, and make sure that each number retrieves the full record of the data. If a new accession number is not linked or a link is broken, provide Spectrum production staff with the correct URL for the record. If the accession numbers for new data are not publicly accessible before the expected online posting of the article, publication may be delayed; please contact production staff (Spectrum@asmusa.org) immediately with the expected release date.

Publication Fees: For information on publication fees and which article types are subject to charges, visit our website. If your manuscript is accepted for publication and any fees apply, you will be contacted separately about payment during the production process; please follow the instructions in that e-mail. Arrangements for payment must be made before your article is published.

ASM Membership: Corresponding authors may join or renew ASM membership to obtain discounts on publication fees. Need to upgrade your membership level? Please contact Customer Service at Service@asmusa.org.

The ASM Journals program strives for constant improvement in our submission and publication process. Please tell us how we can improve your experience by taking this quick Author Survey.

Thank you for submitting your paper to Spectrum.

Sincerely,
Gabriel Parra
Editor
Microbiology Spectrum

Editor Comment:

Please avoid the use of the abbreviation "NoV." Since norovirus is a single word, the NoV abbreviation seems unnecessary.

Reviewer #1 (Comments for the Author):

I have one minor suggestion for the revised manuscript:

Lines 226-228: "Great care was taken to ensure optimal melting temperatures and to avoid secondary structures that could inhibit the isothermal reaction." This sentence is vague and does not convey the actual procedures used to design the primers. Please revise this to include specific details, such as the desired melting temperatures and any tools used to analyze potential secondary structures.

I have one minor suggestion for the revised manuscript:

Lines 226-228: "Great care was taken to ensure optimal melting temperatures and to avoid secondary structures that could inhibit the isothermal reaction." This sentence is vague and does not convey the actual procedures used to design the primers. Please revise this to include specific details, such as the desired melting temperatures and any tools used to analyze potential secondary structures

Point-by-point responses

Comment 1.（Editor Comment）

Please avoid the use of the abbreviation "NoV." Since norovirus is a single word, the NoV abbreviation seems unnecessary.

Response: We are extremely grateful for your thoughtful reminder. All instances of "NoV" have been corrected to "norovirus" in the manuscript, as highlighted in yellow. The correction has also been made in Figure 1B, Supplementary Figure 1, and Tables 1 & 2.

Comment 2.（Reviewer #1）:

I have one minor suggestion for the revised manuscript:

Lines 226-228: "Great care was taken to ensure optimal melting temperatures and to avoid secondary structures that could inhibit the isothermal reaction." This sentence is vague and does not convey the actual procedures used to design the primers. Please revise this to include specific details, such as the desired melting temperatures and any tools used to analyze potential secondary structures.

Response: We deeply appreciate your kind reminder and have now added the specific details of the methods we used (see **Page 9, lines 226-237**) as shown below.

"Based on these alignments, genotype-specific primer sets were designed using SnapGene software (version 2.3.2) (Table 1 and Supplementary Table 1). According to the principle of CPA, target melting temperature (Tm) values were set according to primer function: cross primers (CPR and CPF) and the probe-bearing primer (AMP2) were designed with Tm typically around 65°C to ensure stable target hybridization at the 58°C reaction temperature, while displacement primers (FB and RB) and the internal primer (IP1) were set to Tm values around 55°C, slightly below the reaction temperature, to facilitate strand displacement. To minimize secondary structure–mediated inhibition, primers were screened for hairpin formation and primer-dimer potential using the IDT OligoAnalyzer tool (https://sg.idtdna.com/pages/tools/oligoanalyzer). Notably, the universal stem-loop adapter of the AMP2 probe, which forms an intentional secondary structure, was assessed as part of the intact probe construct rather than as an isolated oligonucleotide. The primers were synthesized by Sangon Biotech (Shanghai, China)."

Re: Spectrum01684-26R2 (**Isothermal Cross-Priming Amplification for Field-Deployable Norovirus Surveillance: A Cost-Effective and Rapid Solution**)

Dear Dr. Jin Xu:

Your manuscript has been accepted, and I am forwarding it to the ASM production staff for publication. Your paper will first be checked to make sure all elements meet the technical requirements. ASM staff will contact you if anything needs to be revised before copyediting and production can begin. Otherwise, you will be notified when your proofs are ready to be viewed.

Data Availability: ASM policy requires that data be available to the public upon online posting of the article, so please verify all links to sequence records, if present, and make sure that each number retrieves the full record of the data. If a new accession number is not linked or a link is broken, provide production staff with the correct URL for the record. If the accession numbers for new data are not publicly accessible before the expected online posting of the article, publication may be delayed; please contact ASM production staff immediately with the expected release date.

Publication Fees: For information on publication fees and which article types have charges, please visit our website. We have partnered with Copyright Clearance Center (CCC) to collect author charges. If fees apply to your paper, you will receive a message from no-reply@copyright.com with further instructions. For questions related to paying charges through RightsLink, please contact CCC at ASM_Support@copyright.com or toll free at +1-877-622-5543. CCC makes every attempt to respond to all emails within 24 hours.

ASM Membership: Corresponding authors may join or renew ASM membership to obtain discounts on publication fees. Need to upgrade your membership level? Please contact Customer Service at Service@asmusa.org.

Embargo Policy: A press release may be issued as soon as the manuscript is posted on the Spectrum Latest Articles webpage. The corresponding author will receive an email with the subject line "ASM Journals Author Services Notification" when the article is available online.

Cover Image Submissions: If you would like to submit a potential Cover Image, please email a file and a short legend to spectrum@asmusa.org. Please note that we can only consider images that (i) the authors created or own and (ii) have not been previously published. By submitting, you agree that the image can be used under the same terms as the published article. Image File requirements: TIF/EPS, 7.5 inches wide by 8.25 inches tall (at least 2,250 pixels wide by 2,475 pixels tall), minimum 300 dpi resolution (600 dpi preferred), RGB, and no figure elements, e.g., arrows or panel labels. The legend should be a short description of the image, 1-2 sentences recommended. Please use the ASM Journals Cover Image Sizing Tool (https://journals.asm.org/pb-assets/html/asm-cover-sizing-tool.html) to ensure that your proposed cover image meets our size requirements.

The ASM Journals program strives for constant improvement in our submission and publication process. Please tell us how we can improve your experience by taking this quick Author Survey.

Thank you for submitting your paper to Spectrum.

Sincerely,
Gabriel Parra
Editor
Microbiology Spectrum
