# Supplementary material for: Isothermal cross-priming amplification for field-deployable norovirus surveillance: a cost-effective and rapid solution
Source: Microbiol Spectr. 2026 Sep 4;14(10):e01684-26. doi: 10.1128/spectrum.01684-26 (PMC13689371; doi:10.1128/spectrum.01684-26)
Supplement: Supplementary file 2 [file spectrum.01684-26-s0001.pdf]

Supplemental material for:

Isothermal Cross-Priming Amplification for Field-Deployable Norovirus Surveillance: A Cost-Effective and Rapid Solution

Ran Jia[1#], Lijuan Lu[1#], Xunhua Zhu[1], Lingfeng Cao[1], Liyun Su[1], Huaqing Zhong[1], Menghua Xu[1], Pengcheng Liu[1], Yanqiong Zhou[2], Jin Xu[1*]

Affiliations:

[1] Department of Clinical Laboratory, Children's Hospital of Fudan University, National Children's Medical Center, Shanghai 201102, China

[2] Ustar Biotechnologies (Hangzhou) Ltd, Hangzhou, Zhejiang 310020, China.

[#] contributed equally as co-first authors.

Address correspondence to:

Jin Xu, PhD., 399 Wanyuan Rd, Minhang, Shanghai 201102, China. E-mail: jinxu_125@163.com. Tel: +86-021-64932947

This file contains one figure (Figure S1) and one table (Table S1).

Table S1 Reaction components of the Lab-CPA system

Components	Final concentration or volume
FB	0.1μM
RB	0.1μM
CPF	0.9μM
CPR	0.9μM
IP1	0.7μM
AMP2	0.1μM
Bst buffer	11.4μL
Bst enzyme	0.27μL
template	5uL
DEPC H2O	up to 35μL

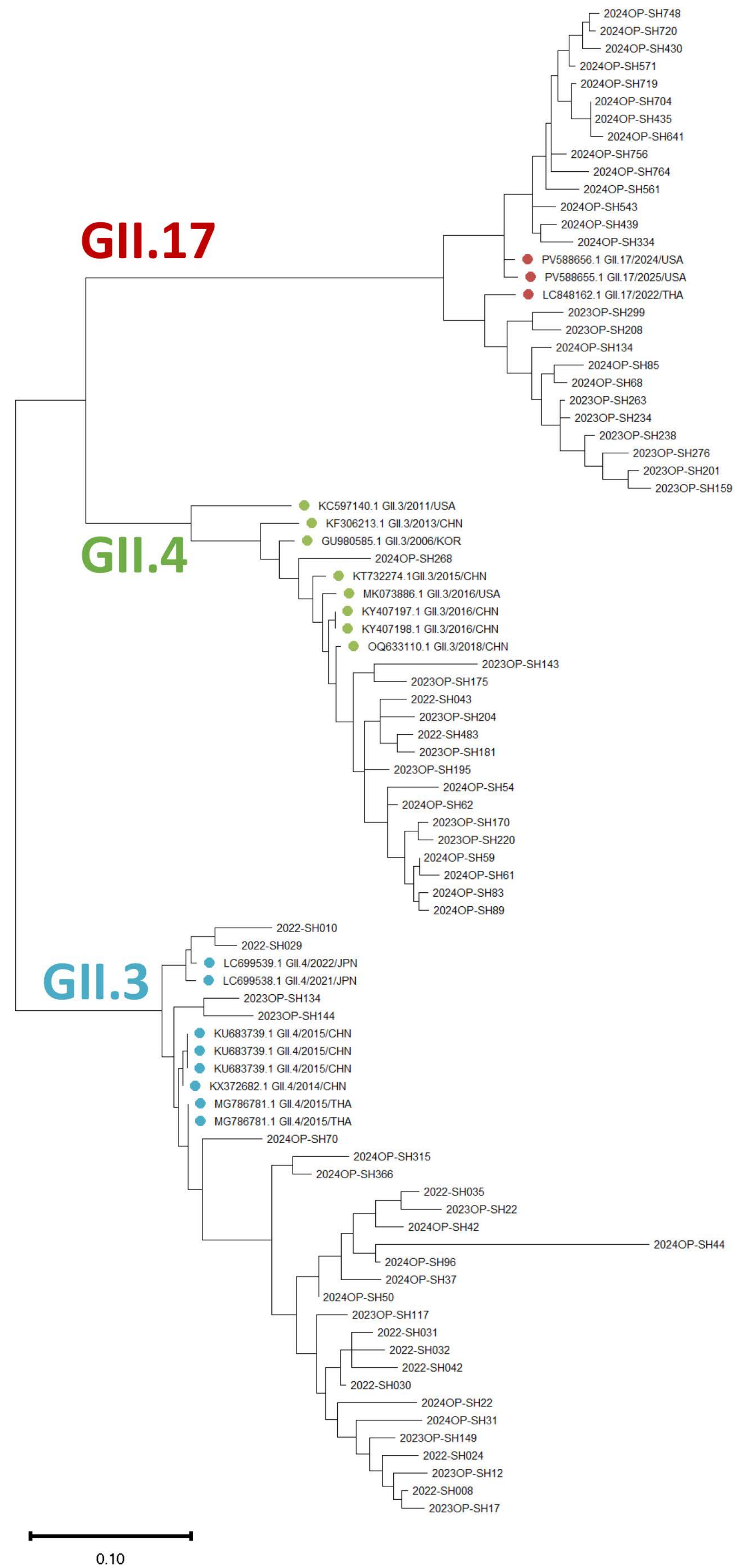

Figure S1 Phylogenetic trees of norovirus strains. The phylogenetic trees of norovirus strains derived in the study were based on the VP1 gene. The phylogenetic trees were constructed using neighbor-joining method with 1,000 bootstrap replicates. Bootstrap values > 70% were shown. Reference genotypes downloaded from the GenBank were labeled with colored dots.
